# Alkaline Bromodeoxyuridine (BrdU) Comet Assay to Detect Replication‐Associated DNA Damage

**DOI:** 10.1002/cpz1.70275

**Published:** 2025-12-11

**Authors:** Diego Luis Ribeiro, James Eduardo Lago Londero, Davi Jardim Martins, Carlos Frederico Martins Menck

**Affiliations:** ^1^ Department of Microbiology, Institute of Biomedical Sciences University of São Paulo São Paulo SP Brazil; ^2^ Chemistry and Environment Center Instituto de Pesquisas Energéticas e Nucleares IPEN‐CNEN São Paulo SP Brazil

**Keywords:** alkaline BrdU comet assay, replicative DNA damage, replication stress, genomic instability, fork progression kinetics

## Abstract

DNA replication is often challenged by endogenous and exogenous sources of DNA damage, which can stall replication forks and result in single‐stranded DNA (ssDNA) gaps, double‐strand breaks (DSBs), and genomic instability. Detecting DNA damage specifically in newly synthesized DNA strands is essential for understanding how eukaryotic cells respond to replication stress and continue the cell cycle progression through DNA repair or DNA damage tolerance (DDT) mechanisms. Here, we present an optimized and accessible protocol for the alkaline BrdU comet assay—a single‐cell technique that combines bromodeoxyuridine (BrdU; a thymidine analog) pulse‐labeling of newly synthesized DNA with the alkaline comet assay (single‐cell gel electrophoresis) followed by fluorescence immunodetection. This method enables the specific detection and measurement of DNA strand breaks occurring in newly replicated DNA during and immediately after the S phase, even without cell synchronization, allowing researchers to differentiate replication‐associated DNA damage from overall genomic damage. We provide detailed instructions for performing the assay using human cells (RPE‐1 h‐TERT TP53 KO) *in vitro* after exposure to DNA replication‐stress‐inducing agents, such as hydroxyurea (HU) and ultraviolet‐C (UV‐C) radiation. We also demonstrate its application in translesion‐synthesis‐deficient (Pol eta‐deficient) human fibroblasts *in vitro*. Importantly, this protocol supports time‐course chase experiments (e.g., 0, 1, 2, and 4 hr post‐treatment) to monitor the kinetics of DNA damage in nascent DNA strands. This BrdU‐based protocol offers high specificity, single‐cell resolution, and cost‐effectiveness, making it particularly valuable for laboratories studying replication stress, post‐replication DNA repair proficiency, DDT, and/or genotoxic responses in unsynchronized human cells *in vitro*. This protocol also adheres to the Minimum Information for Reporting Comet Assay (MIRCA) guidelines and is aligned with the objectives of the International Comet Assay Working Group (ICAW), ensuring high reproducibility and standardization. © 2025 The Author(s). Current Protocols published by Wiley Periodicals LLC.

**Basic Protocol**: Alkaline BrdU comet assay to assess replication‐associated DNA damage in unsynchronized human cells (RPE‐1 h‐TERT TP53 KO) *in vitro*

**Alternate Protocol 1**: Alkaline BrdU comet assay to monitor replication‐associated DNA damage dynamics in unsynchronized human cells (RPE‐1 h‐TERT TP53 KO) after HU and UV‐C exposure and 0‐ to 2‐ hr chase

**Alternate Protocol 2**: Alkaline BrdU comet assay to compare replication‐associated DNA damage dynamics in translesion synthesis polymerase η‐deficient and complemented (XP‐V comp) unsynchronized fibroblasts after UV‐C exposure and 0‐ to 4‐hr chase

## INTRODUCTION

Replication‐associated DNA damage poses a significant risk to genome stability, particularly when eukaryotic cells encounter genotoxic stress during the S phase of the cell cycle (Saxena & Zou, 2022). In these situations, replication forks can stall or collapse, leading to single‐stranded DNA gaps (ssDNA gaps), double‐strand breaks (DSBs), and increased chromosomal instability (Wilhelm et al., 2020). Understanding how human cells respond to these types of damage is crucial for revealing disease mechanisms, particularly in cancer biology (Groelly et al., 2023). The alkaline comet assay (single‐cell gel electrophoresis) is an effective method for detecting DNA strand breaks and alkali‐labile sites (ALS) at the single‐cell level (Collins, 2004; Lu et al., 2017). Although standard alkaline comet assays provide overall DNA damage data, they lack strand specificity and cannot distinguish between parental and newly synthesized DNA strands (Collins et al., 2023). This limitation reduces their usefulness for studies aimed not only at identifying the causative agents or the molecular mechanisms of replication‐associated DNA damage, but also at investigating DNA damage tolerance (DDT) pathways and DNA repair processes, particularly in post‐replicative contexts (Nikolova et al., 2017).

To overcome the limitations of the alkaline comet assay in distinguishing specific replication‐associated DNA damage, this methodology has been adapted to include a bromodeoxyuridine (BrdU) pulse‐labeling step, enabling selective detection of DNA damage in nascent strands. BrdU is a thymidine analog that is incorporated into newly synthesized DNA during the S phase and can be detected explicitly through immunofluorescence (Duque & Rakic, 2015). When combined with the alkaline comet assay, this approach enables precise visualization and quantification of replication‐associated DNA damage—identified as BrdU‐labeled DNA in comet tails—without interference from parental DNA. This refined method, initially introduced by McGlynn et al. (1999) and further optimized by Mórocz et al. (2013), provides a powerful approach for investigating replication stress responses, post‐replication repair (PRR) mechanisms, and gap‐filling efficiency following genotoxic insults. Its single‐cell resolution and specificity, together with the advantage of not requiring cell cycle synchronization, make it particularly valuable for characterizing replication‐associated DNA damage, exploring DDT pathways, and monitoring post‐replicative dynamics in both normal and DNA repair‐deficient cellular contexts.

Here, we present accessible and optimized protocols for high‐resolution single‐cell analysis of replication‐associated DNA damage and repair kinetics in human cells *in vitro*. We employed the RPE‐1 h‐TERT TP53 knockout (KO) cell line, a near‐diploid, non‐transformed retinal epithelial model that avoids the confounding effects of aneuploidy or oncogenic signaling commonly found in cancer cell lines. The *TP53*‐deficient variant enhances detection of replication‐associated lesions (Lambrus et al., 2016) by compromising the DNA damage checkpoint and cell death response, allowing DNA breaks to accumulate and persist—especially in time‐course repair experiments (Castano et al., 2024). To further challenge the assay, we used fibroblasts from patients with xeroderma pigmentosum variant (XP‐V) lacking functional DNA polymerase eta (Polη, encoded by the gene *POLH*), a key enzyme involved in translesion synthesis (TLS) and DDT (Menck & Munford, 2014), as well as *POLH*‐complemented counterparts. Although proficient in lesion removal, XP‐V cells are deficient in DNA synthesis after ultraviolet (UV) radiation and were thus exposed to UV‐C to induce bulky lesions such as cyclobutane pyrimidine dimers (CPDs; Lerner et al., 2017). This setup enabled us to assess replication‐associated DNA damage, PRR efficiency, and gap‐filling capacity in defined genetic contexts, demonstrating the assay's applicability in monitoring genotoxic stress at the single‐cell level.

A key advance of our protocol over earlier versions—such as that described by Mórocz et al. (2013)—is the systematic integration of time‐course experiments (0, 1, 2, and 4 hr post‐treatment), which allows precise kinetic analysis of replication fork progression and PRR in nascent DNA strands. Furthermore, our approach broadens the application to various DNA damage contexts and cellular models, providing flexibility for interrogating dynamic processes such as gap‐filling and post‐replication repair. Compared to conventional alkaline comet assays or γH2AX‐based methods, this protocol offers superior strand specificity, quantitative robustness, and minimal sample preparation requirements. Its affordability, reproducibility, and versatility make it particularly useful for studies involving replication stress, TLS‐ and DNA repair‐deficient syndromes, supporting both mechanistic exploration and therapeutic evaluation in preclinical models. Finally, we report here a first alkaline BrdU pulse comet assay protocol following the Minimum Information for Reporting Comet Assay (MIRCA) guideline (Moller et al., 2020) and in collaboration with the International Comet Assay Working Group (ICAWG), which provides a standardized approach that facilitates reproducibility, inter‐laboratory comparability, and data quality.

*CAUTION*: Biosafety: Perform all procedures involving human cells under Biosafety Level 2 (BSL‐2) conditions, following institutional guidelines.

*CAUTION*: Genotoxicity: Hydroxyurea (HU) and other agents used are potentially mutagenic. Use appropriate personal protective equipment (PPE), including a lab coat, gloves, and eye protection, and handle materials in a certified fume hood or biosafety cabinet.

*NOTE*: BrdU handling: BrdU is light sensitive. Prepare and process BrdU‐containing solutions and slides under low‐light conditions or protect with foil to avoid degradation and preserve antigenicity.

## STRATEGIC PLANNING

**Figure 1:**
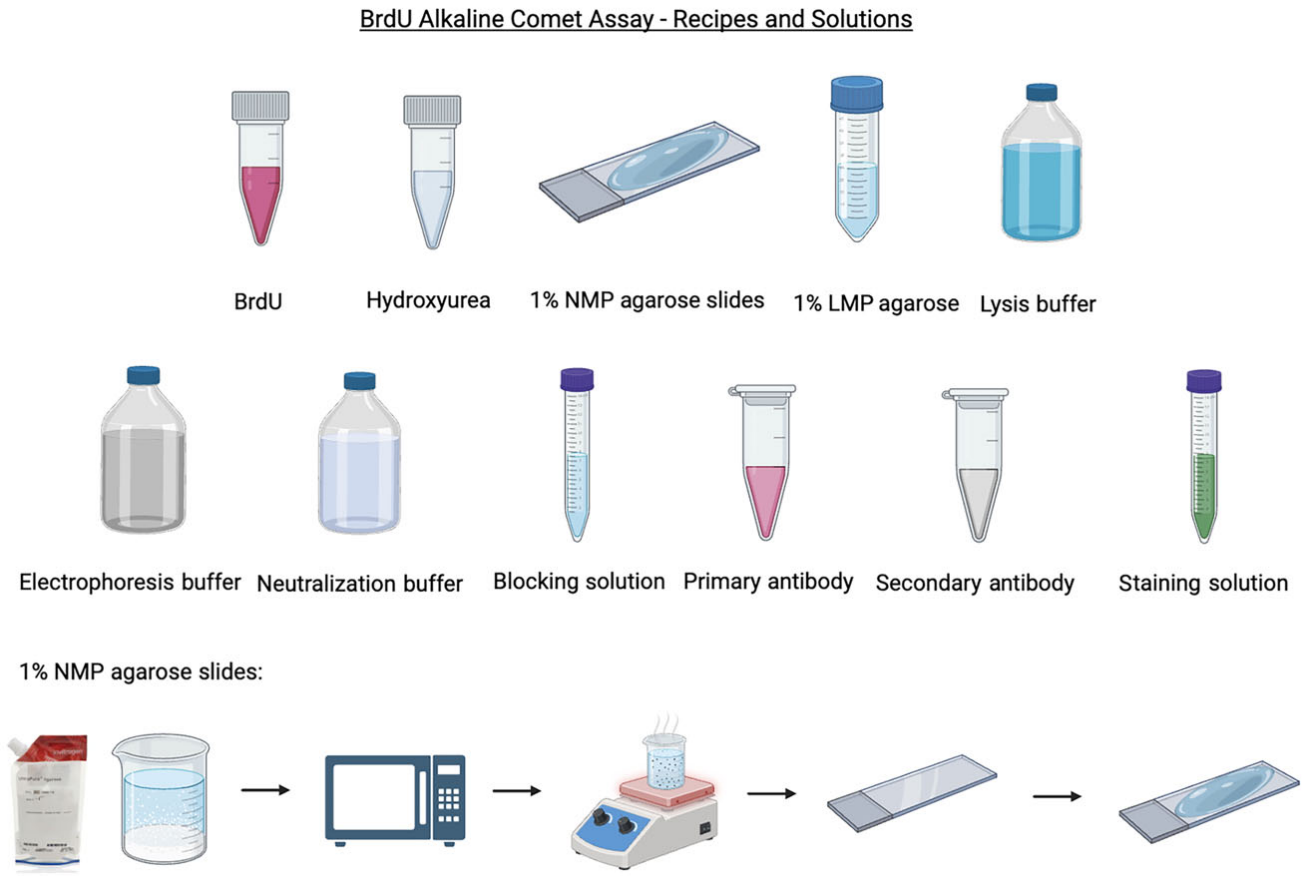
Recipes and solutions for preparing the alkaline BrdU comet assay. Stock solutions (e.g., BrdU, HU) and all buffers (lysis, electrophoresis, neutralization, blocking, and staining) should be prepared in advance. Microscopic slides should be precoated with 1% normal‐melting‐point (NMP) agarose, dried overnight at room temperature, and stored until use. The bottom panel illustrates the workflow for preparing 1% NMP agarose slides, which includes dissolving, heating, temperature equilibration, slide coating, and drying. LMP agarose, low‐melting‐point agarose. Image created with BioRender.

**Figure 2:**
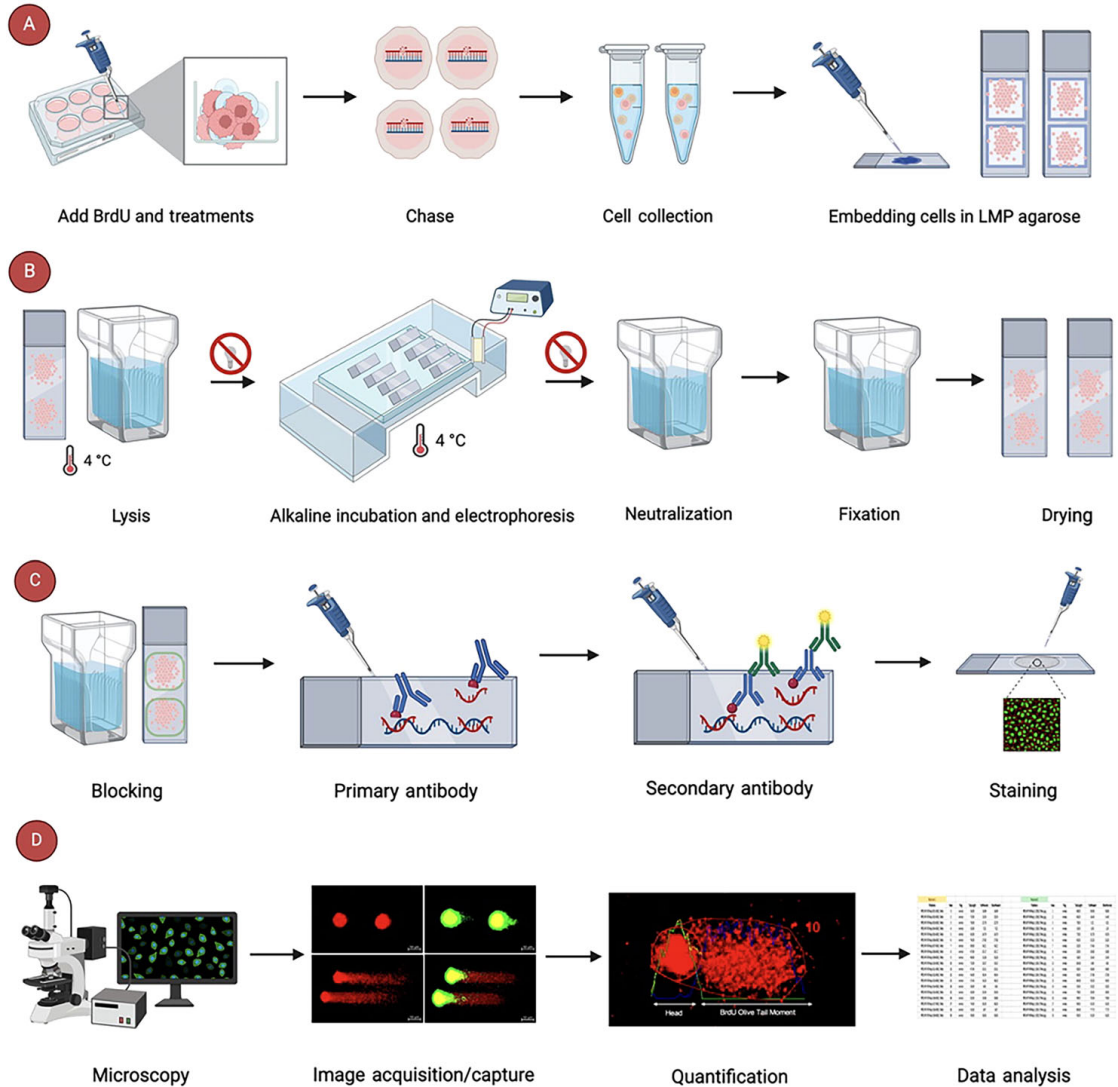
Strategic planning for executing the alkaline BrdU comet assay to analyze replication‐associated DNA damage. Schematic overview: (**A**) Seeding, treatment, and embedding: Cells are pulse‐labeled with BrdU (100 µM) and treated with replication stress agents, followed by an optional chase period. The cells are then harvested and embedded in low‐melting‐point (LMP) agarose on precoated slides. (**B**) Lysis and electrophoresis: Slides undergo lysis, alkaline incubation, and alkaline electrophoresis to facilitate DNA migration. Then, slides are neutralized, fixed, and dried. (**C**) Slides are blocked and incubated with a primary antibody against BrdU, followed by a fluorescent secondary antibody, and then stained. (**D**) Imaging and analysis: Comet images are captured using fluorescence microscopy and analyzed with software or image analysis tools (e.g., OpenComet) to evaluate DNA damage. Image created with BioRender.

This 3‐day protocol requires careful scheduling to reduce variability, especially for replication‐associated DNA damage and post‐replicative consequences in time‐course experiments. See Figures 1 and 2.

**Day 0**: Prepare all stock solutions (i.e., BrdU, HU) and required buffers. Precoat microscopic slides with fresh 1% normal‐melting‐point (NMP) agarose and dry overnight (Fig. 1). Seed cells to reach ∼70%–80% confluency at day 1.

**Day 1**: Treat cells (with HU or other chemicals), along with BrdU (100 µM) pulse‐labeling for 1 hr. Remove treatments/BrdU, replace with fresh culture medium, and incubate (or not) for 1, 2, or 4 hr (“chase” times) before harvesting. For UV‐C (or other radiation types) experiments, expose cells to UV‐C radiation, then do the 1‐hr BrdU pulse‐labeling, followed by the chases. Harvest culture cells and embed in 1% low‐melting (LMP) agarose on precoated 1% NMP agarose slides. Perform lysis, alkaline unwinding, electrophoresis, neutralization, and fixation steps (Fig. 2A and 2B).

**Day 2**: Proceed with BrdU immunostaining. Image and/or analyze comets by fluorescence microscopy and quantify using appropriate software (Fig. 2C and 2D).

## ALKALINE BrdU COMET ASSAY TO ASSESS REPLICATION‐ASSOCIATED DNA DAMAGE IN UNSYNCHRONIZED HUMAN CELLS (RPE‐1 h‐TERT TP53 KO) *IN VITRO*

This protocol demonstrates the use of the alkaline BrdU comet assay to evaluate replication‐related DNA damage at the single‐cell level in human cells *in vitro* without synchronizing the cell culture population. The cells are plated, treated with replication‐stress‐inducing agents, pulse‐labeled with BrdU (during or after the treatment), and then allowed to resolve (or not) the fork collapse/damage in newly synthesized DNA during a specified “chase” period. Next, the cells are harvested, and the comet assay is performed under alkaline conditions. Finally, the DNA with incorporated BrdU (after the cell passes through S phase) is detected via immunofluorescence. When done correctly, users can visualize and measure DNA strand breaks specifically in newly synthesized DNA, which appear as BrdU‐labeled comet tails. To demonstrate the versatility and sensitivity of this method, we employed the RPE‐1 h‐TERT TP53 KO cells. The *TP53* deletion enhances the retention of cells with DNA damage, thereby increasing the detection of replication‐associated lesions without introducing significant genomic instability. This makes RPE‐1 TP53 KO cells especially useful for analyzing DNA damage events during S‐phase progression. Overall, this assay provides a sensitive, cost‐effective, and versatile approach for investigating replication stress, PRR mechanisms, DDT pathways, and the dynamics of replication‐associated DNA damage.

### Materials

Milli‐Q‐purified water (MilliporeSigma)
1% (w/v) normal‐melting‐point (NMP) agarose precoating solution (prepared fresh by dissolving UltraPure NMP Agarose [Invitrogen, cat. no. 16500500] in Milli‐Q water and heating until fully dissolved)
5‐Bromo‐2′‐deoxyuridine (BrdU; TargetMol, cat. no. T6794, CAS no. 59‐14‐3)
Dimethyl sulfoxide (DMSO; Sigma‐Aldrich, cat. no. D5879‐1L)
Hydroxyurea powder (HU; Sigma‐Aldrich, cat. no. H8627)
Sodium chloride (NaCl; Sigma‐Aldrich, cat. no. 71380‐1KG)
Tris base (Sigma‐Aldrich, cat. no. T1503‐500G)
Ethylenediaminetetraacetic acid disodium salt dihydrate (C_10_H_14_N_2_Na_2_O_8_·2H_2_O, EDTA‐Na_2_·2H_2_O; Merck KGaA, cat. no. E5134) or 500 mM EDTA solution, pH 8.0 (TekNova, cat. no. E0308; 1000 ml)
Sodium hydroxide (NaOH; Merck, cat. no. 1.06498.100)
RPE‐1 h‐TERT TP53 knockout (KO) cells: TERT‐immortalized human retinal pigment epithelial cells (ATCC CRL‐4000, with TP53 knockout generated by CRISPR‐Cas9; kindly provided by Dr. Stephen Jackson (University of Cambridge, Cancer Research UK Institute, and available upon reasonable request to the corresponding author, subject to authorization from Dr. Jackson)
DMEM/F‐12 medium (ATCC, cat. no. 30‐2006; 2.5 mM l‐glutamine, 15 mM HEPES, 0.5 mM sodium pyruvate, and 1200 mg/L sodium bicarbonate; 500 ml)
Fetal bovine serum (FBS), qualified, heat inactivated (Gibco, cat. no. 16140071; 500 ml)
100× antibiotic‐antimycotic mix (Gibco, cat. no. 15240062; 10,000 units/ml penicillin, 10,000 µg/ml streptomycin, and 25 µg/ml amphotericin B; 100 ml)
PBS (1×), pH 7.4, without calcium and magnesium (Quality Biological, cat. no. 114‐058‐101)
TrypLE Select enzyme (1×; Gibco, cat. no. 12563029; phenol red‐free; 500 ml)
Low Melting Point (LMP) Agarose (Invitrogen, cat. no. 16520050)
70% (v/v) or 99% ethanol: ethyl alcohol, pure ≥99.5%, anhydrous, 200 proof (Sigma‐Aldrich, cat. no. 459836‐1L), diluted appropriately or undiluted, as preferred (see step 43)
Primary antibody solution: anti‐BrdU monoclonal (clone B44; see recipe; suggested concentration 2.5 µg/ml in 1% BSA/PBS)
Secondary antibody solution: anti‐mouse secondary antibody conjugated to Alexa Fluor 594 (see recipe; suggested concentration 50 µg/ml in 1% BSA/PBS)
Blocking buffer (see recipe; prepared fresh)
0.1% PBS‐T (see recipe)
SYBR Green staining solution (see recipe; prepare fresh on day of use)
Optional: 0.4% trypan blue stain (Invitrogen, cat. no. T10282)

Superfrost Plus microscope slides (ThermoScientific, cat. no. J1800AMNZ)
0.6‐ml microcentrifuge tubes (Phytotech Lab, cat. no. C1992)
1.5‐ml microcentrifuge tubes, sterile (Eppendorf, cat. no. 0030120086)
0.5‐ to 10‐µl, 2‐ to 20‐µl, 20‐ to 200‐µl, and 100‐ to 1000‐µl micropipets (Eppendorf or equivalent)
Humidified 37°C, 5% CO_2_ tissue culture incubator
Hemacytometer (e.g., Neubauer chamber) or automated cell counter (e.g., Countess II (Life Technologies, cat. no. AMQAf1000) and Countess cell counting chamber slides (Invitrogen, cat. no. 10228)
6‐well clear TC‐treated multiple well plates (polystyrene, flat bottom, sterile; Corning, cat. no. CLS3516)
Serological pipets
Microcentrifuge (standard microcentrifuge for 1.5‐ to 2‐ml tubes, capable of reaching 300 × *g*)
Coplin jars (able to hold 5‐10 microscope slides)
Aluminum foil: generic, laboratory‐grade for light protection
Comet Assay Electrophoresis System (e.g., Trevigen, cat. no. 4250‐050‐ES; equivalent systems from other manufacturers may be used if compatible with the assay protocol)
Electrophoresis power supply (e.g., Bio‐Rad Powerpack HC Power Supply or equivalent)
Hydrophobic barrier pen: PAP Pen Liquid Blocker (Sigma‐Aldrich, cat. no. Z377821)
22 × 22‐mm rectangular coverslips (Thermo Scientific, cat. no. MA062210)
EVOS FL Auto 2 Imaging System 5000 (Thermo Fisher Scientific, cat. no. AMAFD2000)
Fluorescence microscope (EVOS FL Auto 2 Imaging System, EVOS M5000 Fluorescence Microscope, or equivalent system)
Humid chamber: black plastic box with a lid large enough to hold 10‐12 slides on a wet paper towel and that fits in the 37°C incubator (e.g., a slide staining tray with a black lid designed for immunohistochemistry, or a microscopic slide box covered with aluminum foil)
Styrofoam box or similar to accommodate solutions in ice
Comet assay analysis software (e.g., Comet Assay IV, Comet Score, CASP, Komet, or equivalent)
Optional: Fiji (ImageJ; Schindelin et al., 2012) with OpenComet plugin (Gyori et al., 2014; free download available from https://imagej.net/Fiji and http://www.cometbio.org/)
GraphPad Prism v. 10.0 or later
Microsoft Excel (Microsoft 365)

#### Day 0: Coating microscopic slides, preparing solutions, and cell culture seeding

1 Prepare microscope slides coated with fresh 1% NMP agarose (see recipe) and allocate at least one slide per treatment condition.
  This is best done on the day before the experiment to ensure optimal adherence and performance.
2 Prepare the following solutions (see recipes in Reagents and Solutions): BrdU stock and working solutions, HU stock solution, lysis stock solution, electrophoresis buffer solutions A and B, and neutralization buffer.
3 Culture RPE‐1 h‐TERT TP53 KO cells in DMEM/F12 medium supplemented with 10% FBS and 1% antibiotic‐antimycotic mix (complete culture medium) in a humidified 5% CO_2_, 37°C incubator.
4 When the cells reach exponential growth phase at 70%‐80% confluency, remove the culture medium from the flask.
5 Rinse the cell monolayer with sterile 1× PBS (pH 7.2‐7.4) to remove residual serum.
6 Add 1× TrypLE Express (or equivalent trypsin) and incubate at 37°C until cells detach.
7 Inactivate the TrypLE solution by adding 3 vol of complete culture medium.
8 Determine cell density using a hemacytometer (e.g., Neubauer chamber) or an automated cell counter (e.g., Countess).
9 Seed 0.2 × 10^6^ cells per well in a six‐well plate with 2 ml of complete culture medium.
10 Incubate for 24 hr in a 37°C, 5% CO_2_ tissue culture incubator to allow cell culture stabilization (reattachment and physiological activity).

#### Day 1: Treatments, BrdU labeling, chase time, and embedding cells

11 Add BrdU working solution to all wells to obtain a final BrdU concentration of 100 µM.
12 Add HU stock solution to the appropriate wells to obtain a final HU concentration of 400 µM. Gently swirl the plate to ensure an even distribution.
13 Replace the plate in the tissue culture incubator and incubate it for 1 hr.
14 Remove the culture medium with a serological pipet.
15 Wash the wells once with 1× PBS (1‐2 ml). Carefully remove the PBS using a serological pipet.
16 Add fresh complete culture medium (1‐2 ml), replace the plate in the 37°C, 5% CO_2_ tissue culture incubator, and incubate for the chase period (1‐2 hr).
  The chase time is variable depending on the hypothesis being tested. If you do not need to perform a chase, proceed directly to harvest.
17 To harvest cells, first remove the culture medium using a serological pipet.
18 Wash once with 1× PBS to remove residual debris. Remove the PBS using a serological pipet.
19 Detach cells by adding 1× TrypLE Express (∼250 µl) and incubating at 37°C for 3‐5 min.
20 Inactivate the TrypLE solution by adding 3 vol (∼750 µl) complete culture medium.
21 Carefully mix the well contents 5‐10 times by washing the well sides to loosen and break up lumps.
22 Collect the cell suspension in 1.5‐ml microcentrifuge tubes and centrifuge 5 min at 300 × *g*, at room temperature.
23 Discard most of the supernatant, leaving 100 µl in the tube, and gently resuspend the pellet in the remaining culture medium.
24 Place the tubes on ice to keep the cells cold.
25 Prepare a workstation with slides precoated with 1% NMP agarose and labeled appropriately for each condition to be tested. Prepare the 1% LMP agarose (see recipe) and keep at ∼37°C (ensure that the agarose is fully dissolved).
26 In a fresh 1.5‐ml centrifuge tube, place 90 µl of 1% LMP agarose.
27 Add 10 µl of the cell suspension to the LMP agarose (representing a 1:10 [v/v] dilution) and mix gently.
28 Dispense a 50‐µl drop of the mixture onto each 1% NMP agarose‐precoated slide in two separate drops.
29 Cover drops with 22 × 22‐mm coverslips and place slides at 4°C for at least 30 min to allow the agarose to solidify.

#### Lysis, alkaline treatment, electrophoresis, neutralization, and fixation

30 Prechill the lysis working solution (prepared in step 2) at 4°C.
31 Carefully remove the coverslips from the drops. Verify that the LMP agarose is wholly solidified without damage to the cells. Once solidification has occurred, proceed to the lysis step.
32 Place the comet slides in a black Coplin jar or similar slide holder. If a clear Coplin jar is used, wrap it in aluminum foil to prevent exposure to light.
33 Submerge the slides in ice‐cold lysis working solution and incubate for 1 hr at 4°C.
  If necessary, slides can stay in lysis solution overnight at 4°C; see protocol variation point 5 in Critical Parameters.
34 Prepare the alkaline electrophoresis buffer (0.3 M NaOH/1 mM EDTA, pH 13) by adding solution A (10 M NaOH) and solution B (200 mM EDTA), prepared in step 2, to Milli‐Q water prechilled at 4°C.
35 Wash the slides by dipping them into a Coplin jar containing prechilled Milli‐Q water.
36 Place the slides in an electrophoresis chamber filled with the ice‐cold alkaline electrophoresis buffer (make sure the chamber is leveled with the bench using a spirit level before adding the buffer).
37 Incubate the slides for 25 min in the ice‐cold electrophoresis buffer (alkaline treatment).
  Avoid excessive incubation to prevent degradation; see protocol variation point 6 in Critical Parameters.
38 Conduct an electrophoresis run, preferably at a voltage of 1 V/cm (25 V in total) for 25 min. Maintain a consistent buffer temperature (4°C‐10°C) to ensure uniform DNA unwinding. If necessary, use an ice bath or cooling system.
39 Carefully remove the slides from the electrophoresis chamber and place them on a vertical rack to drain.
40 Transfer the comet slides into an empty Coplin jar or horizontal slide holder.
41 Gently immerse the slides in neutralization buffer until they are completely covered.
  If buffer is not available, 1× PBS and distilled water can be used as alternatives; see protocol variation point 7 in Critical Parameters.
42 Wash the slides three times for 5 min each with neutralization buffer (prepared in step 2). After the final washing, remove the slides and place them on a rack or a slide holder to drain at room temperature.
43 Transfer the slides to a Coplin jar and fix them by immersing them in 70% (v/v) ethanol for 5 min.
  Alternatively, 99% ethanol can be used to maintain the comets at the same level; see protocol variation point 8 in Critical Parameters.
44 Remove the slides and place them on a vertical rack to dry completely in the air at room temperature.
  The slides can be stored covered overnight at room temperature.

#### Day 2: Immunostaining

45 Calculate the required volumes for the following solutions based on slide number: (i) blocking buffer (∼300‐500 µl per slide); (ii) anti‐BrdU primary antibody (1:100 dilution; ∼30‐60 µl per slide); (iii) anti‐mouse secondary antibody conjugated to Alexa Fluor 594 (1:250 dilution; ∼50‐100 µl per slide).
46 To maintain humidity during antibody incubations, use an appropriate horizontal slide holder as a humid chamber (e.g., a slide staining tray with a black lid for immunohistochemistry) or a microscopic slide box covered with aluminum foil.
47 To restrict reagents, outline agarose areas using a hydrophobic barrier pen. Allow the markings to dry for 1‐2 min.
48 Wash the comet slides in 1× PBS using a Coplin jar.
49 Using a micropipet, cover the agarose area with (at least) 300 µl blocking solution and incubate for at least 30 min at room temperature.
50 Remove the blocking solution. Wash the slides once with 0.1% PBS‐T and allow them to dry at room temperature.
51 Place the comet slides back in the dark humid chamber. If necessary, re‐outline the agarose area with the hydrophobic pen.
52 Using a micropipet, apply a drop (30‐60 µl) of the primary antibody solution (mouse anti‐BrdU; 2.5 µg/ml in 1% BSA/PBS) to each agarose area.
53 Gently place a coverslip over the drop, ensuring the solution spreads evenly.
54 Incubate in the dark humid chamber at room temperature for 1 hr.
55 Carefully remove the coverslip using tweezers and place the slides in a Coplin jar to remove excess primary antibody.
56 Wash slides three times with 0.1% PBS‐T. After drying, return the comet slides to the dark humid chamber.
57 Using a micropipet, apply a drop (50‐100 µl) of secondary antibody solution (goat anti‐mouse IgG Alexa Fluor 594 conjugate; 1:250 dilution in 1% BSA/PBS) to each agarose area.
58 Gently place a coverslip over the drop to ensure that the solution spreads evenly.
59 Incubate in the dark at room temperature for 1 hr.
60 Carefully remove the coverslip using tweezers.
61 Wash the slides in 0.1% PBS‐T using a Coplin jar to remove excess secondary antibody. Perform three washes to ensure thorough removal of the stain.
62 After the final washes, allow the slides to drain and air‐dry completely at room temperature.
63 Apply (50‐100 µl) SYBR Green staining solution drop directly onto each agarose area. Incubate for 10‐15 min in the dark.
  This step is optional when analyzing overall genomic DNA damage (not associated with replication).
64 Carefully remove the excess SYBR stain and wash slides once with Milli‐Q water. Slides can be imaged immediately or stored in a dark chamber (or microscopy box) until analysis. Work with minimal light exposure.

#### Microscopy, image acquisition, and data analysis

65 Using a slide holder, place the stained comet slides on the fluorescence microscope stage, with the agarose gel side facing the objective lens.
66 Observe comets under a 10× objective. Use the Red channel (Texas Red) to visualize BrdU‐labeled nucleoids and comet tails.
67 Randomly select fields of view on each slide, avoiding edges, air bubbles, and damaged agarose areas. Orient comets horizontally, with the head on the left and the tail extending to the right.
68 Capture and save images of randomly selected comets per slide in the red channel (BrdU staining). Additional photos can be taken in the green (SYBR) and merged (SYBR + BrdU) channels if desired. Save in TIFF or JPEG format with a bright DNA signal on a dark background.
69 For image analysis, use commercial comet analysis software to quantify parameters such as Olive tail moment (OTM), % tail DNA, and tail length across the fluorescence channels.
70 If commercial software is unavailable, use the OpenComet plugin in ImageJ/Fiji as an open‐source alternative for single‐channel or basic dual‐channel analysis. Ensure that all detection parameters are optimized for consistent quantification of tail DNA.
71 Analyze at least 50 comets per gel (or 100 per sample for duplicates). Store raw and processed data for subsequent statistical analysis. Always include a negative control (e.g., 1× PBS or 1% DMSO) and a known positive control (e.g., HU‐treated sample) to confirm assay performance.

## Alternate Protocol 1 ALKALINE BrdU COMET ASSAY TO MONITOR REPLICATION‐ASSOCIATED DNA DAMAGE DYNAMICS IN UNSYNCHRONIZED HUMAN CELLS (RPE‐1 h‐TERT TP53 KO) AFTER HU AND UV‐C EXPOSURE AND 0‐ TO 2‐HR CHASE

This protocol is designed to investigate the early dynamics of replication‐associated DNA damage under conditions of chemical and radiation‐induced stress. By combining BrdU pulse‐labeling with short chase periods, it enables the detection of how newly synthesized DNA is affected by replication fork stalling (HU) or photoproducts (UV‐C). Monitoring these early timepoints (0‐2 hr) provides information on the persistence of DNA strand discontinuities, their initial processing, and the capacity to stabilize or resolve replication stress. This approach is particularly valuable for comparing the distinct types of replication‐associated DNA lesions caused by different genotoxic agents and for determining whether cells can efficiently tolerate or repair DNA damage.

### Additional Materials (also see Basic Protocol)

UV‐C light source (254 nm) calibrated with a dosimeter (VLX‐3W radiometer)
UV‐protective eyewear and shielding

Perform all steps described in the Basic Protocol, with the following modifications:

1 In steps 11 and 12, perform the desired treatments:
  a. HU: Add 100 µM BrdU and 400 µM HU to the cells.
  b. UV‐C: Remove culture medium from the cells, rinse the cells once with 1× PBS, and irradiate them in 1 ml of 1× PBS at 10‐30 J/m^2^. Remove the 1× PBS and add 100 µM BrdU in fresh complete culture medium. Place the plate in culture incubator for 1 hr.
2 After the 1‐hr BrdU pulse, incubate cells in fresh complete culture medium for 1 or 2 hr (chase) in a 37°C, 5% CO_2_ incubator. If a no‐chase‐point condition (0‐hr) is the objective, proceed to step 17 of the Basic Protocol. Use a timer to ensure precise intervals.
3 With the groups of cells from each time point, proceed with Basic Protocol, steps 17‐71, to harvest and collect cells and continue with the comet assay (cell embedding, lysis, alkaline treatment, electrophoresis, neutralization, fixation, immunostaining, image acquisition, and analysis).
  When analyzing comet assay results, the Olive tail moment (OTM) quantifies the extent of DNA damage in individual cells. It is expressed in arbitrary units (A.U.) and integrates both the amount of DNA present in the comet tail (reflecting the fraction of damaged DNA) and the tail length (indicating the severity of DNA fragmentation). Therefore, higher OTM values correspond to longer tails and greater levels of DNA strand breaks (Collins, 2004).
  0 hr (no chase): HU‐treated cells show distinct BrdU‐positive comet tails, indicating immediate replication‐related strand breaks caused during treatment. UV‐C‐exposed cells require higher doses to produce more noticeable BrdU comet tails.
  1‐2 hr chase: In HU‐treated cells, replication‐associated DNA damage often persists due to fork stalling and collapse; in UV‐C‐induced damage, it typically shows a more evident increase during early chase.
  Expected trend: BrdU olive tail moment values are higher in HU‐treated cells. The values in UV‐C–treated cells increase over time during the chase. However, recovery mechanisms, including PRR and TLS, may contribute to partial resolution.

## Alternate Protocol 2 ALKALINE BrdU COMET ASSAY TO COMPARE REPLICATION‐ASSOCIATED DNA DAMAGE DYNAMICS IN TRANSLESION SYNTHESIS POLYMERASE η‐DEFICIENT (XP‐V) AND COMPLEMENTED (XP‐V comp) UNSYNCHRONIZED FIBROBLASTS AFTER UV‐C EXPOSURE AND 0‐ TO 4‐HR CHASE

This protocol uses the alkaline BrdU comet assay to monitor replication‐associated DNA damage in SV40‐transformed human fibroblasts derived from an XP‐V patient (XP30RO). XP‐V cells lack functional TLS DNA polymerase eta (Polη; *POLH* ^−^/^−^ genotype), while their complemented counterparts (XP‐V Comp; XP30RO clone 6, with wild‐type *POLH* reintroduced) have restored TLS activity (Stary et al., 2003; the two cell lines were generously provided by Dr. Anne Stary and Dr. Patricia Kannouche, Institut Gustave Roussy, Villejuif, France). After UV‐C irradiation, these fibroblasts accumulate bulky lesions such as cyclobutane pyrimidine dimers (CPDs), which block replication forks. Cells are then pulse‐labeled with BrdU, and chase periods of 0, 1, 2, or 4 hr after treatment allow the evaluation of how DNA damage kinetics are resolved over time, providing a direct measure of PRR efficiency in TLS‐deficient versus TLS‐proficient cells.

### Additional Materials (also see Basic Protocol)

XP‐V (XP30RO) fibroblasts: SV40‐transformed fibroblasts derived from an XP‐V patient, deficient in DNA polymerase η (Polη; *POLH* ^−^/^−^)
XP‐V Comp (XP30RO complemented fibroblasts): SV40‐transformed fibroblasts derived from the same XP‐V patient, reconstituted with wild‐type *POLH* to restore TLS Polη function

UV‐C light source (254 nm) calibrated with dosimeter (VLX‐3W radiometer)
Fibroblast culture medium: DMEM High‐Glucose + 10% FBS + 1% antibiotic/antimycotic
UV safety shielding

Perform all steps described in the Basic Protocol, with the following modifications specific to XP‐V and XPV‐Comp cells.

1 In step 3, grow XP‐V and XP‐V Comp cells separately to 70%‐80% confluence. Seed 0.2 × 10^6^ of each cell line into separate wells of 6‐well plates and incubate for 24 hr in a 37°C, 5% CO_2_ incubator.
2 Remove the medium and add 1 ml 1× PBS to each plate well as described in the Basic Protocol, step 5.
3 Remove plate lid and irradiate the cells with UV‐C (10 or 30 J/m^2^).
4 Remove 1× PBS, add fresh complete culture medium containing 100 µM BrdU (pulse label), and incubate for 1 hr in the 37°C, 5% CO_2_ incubator.
5 Immediately after the 1 hr incubation, remove the medium and add BrdU‐free complete culture medium to start the chase period. For no‐chase samples, harvest the cells and proceed to step 8 below.
6 Incubate the cells for 1, 2, or 4 hr (for the successive time points) in a 37°C, 5% CO_2_ incubator.
7 At each time point, harvest cells by trypsinization as described in the Basic Protocol, steps 17‐24.
8 Prepare comet slides and perform lysis, alkaline electrophoresis, neutralization, and fixation as described in the Basic Protocol, steps 25‐45.
9 Perform BrdU immunostaining as described in the Basic Protocol, steps 46‐65.
10 Capture the images and quantify the comets as described in the Basic Protocol, steps 65‐71.
  0‐hr chase: XP‐V and XP‐V Comp fibroblasts exhibit BrdU‐positive comet tails immediately after UV‐C exposure, especially at higher doses, indicating replication‐associated strand breaks at UV‐induced lesion sites.
  1‐4 hr chase: In XP‐V cells, BrdU OTM is expected to increase further, as replication forks progressively encounter unrepaired CPDs and undergo stalling in the absence of Polη‐mediated TLS. XP‐V cells exhibit persistent damage at 1‐4 hr, indicating impaired TLS bypass of CPDs. In XP‐V Comp cells, the comet tails are also detected; the increase over time is less pronounced. The functional TLS Polη facilitates lesion bypass and PRR, leading to attenuated damage accumulation and partial resolution.
  Overall interpretation: XP‐V cells exhibit sustained or even increased replication‐associated DNA damage during the chase period, whereas XP‐V comp cells show more regulated dynamics, emphasizing the critical role of TLS in managing UV‐induced replication stress. This protocol can be adapted for other TLS‐deficient or DNA repair‐deficient models and combined with pharmacological inhibitors to analyze specific pathway contributions.

## REAGENTS AND SOLUTIONS

### Alkaline electrophoresis buffer (working solution), pH 13

**Table:** 

Reagent	Final concentration	Quantity or volume
Electrophoresis solution A (10 M NaOH; see recipe)	0.3 M	60 ml
Electrophoresis solution B (200 mM EDTA; see recipe)	1 mM	10 ml
Milli‐Q‐purified (ultrapure) water, 4°C	–	1930 ml
Total		2000 ml

Prepare fresh on the day of use and use within 1‐12 hr. Chill Milli‐Q H_2_O at 4°C at least 1 day before. On the day of the assay, mix 1930 ml of chilled Milli‐Q H_2_O with 40 ml of electrophoresis solution A (10 M NaOH) and 30 ml of electrophoresis solution B (200 mM EDTA, pH 10). Stir and check the pH using a pH meter accurate to 0.01 unit; adjust to pH 13, if necessary, by adding more solution. Keep the buffer at 4°C until the electrophoresis is performed. Usually, 2 L is enough for 1‐2 runs, depending on the tank used.

### Blocking buffer (1% BSA/PBS)

**Table:** 

Reagent	Final concentration	Quantity or volume
Bovine serum albumin (BSA; Sigma‐Aldrich, cat. no. A9418)	1% (w/v)	0.1 g
1× PBS, pH 7.4		10 ml
Total		10 ml

Prepare fresh on the day of use and store at room temperature for up to 1 day. In ∼40 ml of 1× PBS, dissolve 0.5 g of BSA with continuous stirring until fully dissolved (5‐10 min). Adjust the final volume to 50 ml with 1× PBS. Use immediately; discard any leftover solution after 24 hr.

### BrdU stock solution, 100 mM

**Table:** 

Reagent	Final concentration	Quantity or volume
5‐Bromo‐2′‐deoxyuridine (BrdU)	100 mM	10 mg
Dimethyl sulfoxide (DMSO)	–	0.3256 ml
Total	–	0.3256 ml

Remove the BrdU powder from the freezer (–20°C) and let it reach room temperature before opening. Use a micropipet to add DMSO, then gently mix until fully dissolved, ensuring no clumps remain. Aliquot the solution (e.g., 10‐20 µl) into sterile, labeled tubes. Protect from light during both preparation and storage. Prepare at least 1 day before use. Store aliquots up to 1 month at –20°C or up to 1 year –80°C (for long‐term storage).

### BrdU working solution, 10 mM

**Table:** 

Reagent	Final concentration	Quantity or volume
BrdU stock solution (100 mM)	10 mM	20 µl
DMSO	–	180 µl
Total	–	200 µl

This 100 × working solution is used at 1% (v/v) to achieve a final concentration of 100 µM BrdU in the culture medium. Prepare at least 1 day before use. Thaw a 100 mM BrdU stock aliquot on ice, and in a sterile 1.5‐ml tube, mix it with DMSO by pipetting (avoid vortexing). Keep sterility and protect from light. Store up to 1 year at –80°C.

### Electrophoresis buffer solution A (200 mM EDTA)

**Table:** 

Reagent	Final concentration	Quantity or volume
Ethylenediaminetetraacetic acid (EDTA)	200 mM	14.89 g
Milli‐Q‐purified (ultrapure) water	–	∼ 150 ml (adjust to 200 ml)
Total		200 ml

Prepare at least 1 day before use. Store the solution at room temperature for 7‐15 days. Add Milli‐Q H_2_O to a glass flask on a magnetic stirrer. Gradually add EDTA disodium salt while stirring. Add solid NaOH slowly in small portions to adjust the pH to 10, using a pH meter accurate to 0.01 unit. Once fully dissolved, transfer the solution to a graduated cylinder, bring the volume to 200 ml with Milli‐Q water, and store it in a clean, labeled bottle.

*Alternative method (using premade 500 mM EDTA, pH 8.0)*: Mix 80 ml of the EDTA stock with 120 ml of Milli‐Q H_2_O while stirring. Adjust the pH to 10 by adding NaOH dropwise. Adjust the final volume to 200 ml. Transfer to a clean bottle for storage.

### Electrophoresis buffer solution B (10 M NaOH)

**Table:** 

Reagent	Final concentration	Quantity or volume
Sodium hydroxide (NaOH)	10 M	100 g
Milli‐Q‐purified (ultrapure) water	–	250 ml
Total		250 ml

Prepare at least 1 day before use. Wear PPE and work inside a fume hood. Add ∼200 ml of Milli‐Q H_2_O to a 500‐ml heat‐resistant glass flask on a magnetic stirrer. Gradually add NaOH in small portions (note: this is an exothermic reaction). After complete dissolution and cooling, transfer the solution to a 250‐ml cylinder and top it up with Milli‐Q H_2_O. Store in a labeled bottle at room temperature for up to 15 days. Avoid overheating to prevent splashing and damage to the glassware.

### Hydroxyurea stock solution, 40 mM

**Table:** 

Reagent	Final concentration	Quantity or volume
Hydroxyurea (HU) powder	40 mM	10 mg
Milli‐Q‐purified (ultrapure) water, sterile	–	2.1038 ml
Total	–	2.1038 ml

Prepare at least 1 day before use to ensure complete dissolution. Weigh 10 mg of HU and dissolve it in sterile Milli‐Q H_2_O in a 15‐ml sterile tube. Mix gently by pipetting until the solution is fully dissolved. Aliquot 100‐200 µl into sterile 1.5 ml microtubes. Protect from light. Store up to 1 month at –20°C (short‐term storage) or up to 1 year at –80°C (long‐term storage).

### Hydroxyurea working solution, 400 µM

**Table:** 

Reagent	Final concentration	Quantity or volume
Hydroxyurea stock solution (40 mM)	400 µM	10 µl
Cell culture medium	–	990 µl
Total	–	1000 µl

Prepare fresh on the day of the assay. Thaw a 40 mM HU stock aliquot on ice, protected from light. Dilute 1% (v/v; 1:100 dilution) directly into complete culture medium to achieve a final concentration of 400 µM (for example, 10 µl HU stock + 990 µl medium). Maintain sterility and prevent contamination by using clean, sterile tips and tubes.

### Low‐melting‐point (LMP) agarose, 1% (for cell embedding)

**Table:** 

Reagent	Final concentration	Quantity or volume
UltraPure LMP Agarose (Invitrogen, cat. no. 16520050)	1% (w/v)	0.1 g
Milli‐Q‐purified (ultrapure) water	–	10 ml
Total	–	10 ml

Prepare fresh just before the assay. Weigh LMP agarose, dissolve it in Milli‐Q H_2_O using a microwave (swirl every 20‐30 s until transparent), and keep at 37°C in a water bath to prevent solidification. Use the embedded cells immediately on precoated slides. Do not reuse leftovers. Avoid overheating and work quickly to prevent premature gelling.

### Lysis stock solution

**Table:** 

Reagent	Final concentration	Quantity or volume
Sodium chloride (NaCl)	2.5 M	146.1 g
Ethylenediaminetetraacetic acid (EDTA)	100 mM	37.2 g
Tris base	10 mM	1.2 g
Milli‐Q‐purified (ultrapure) water	–	∼850 ml (adjust to 1 liter)
Total	–	1000 ml

In a 1‐liter beaker containing Milli‐Q H_2_O, add the following components sequentially: NaCl, EDTA, and Tris base. Stir for ∼20 min, or until the mixture is fully dissolved. Adjust the pH to 10.0 by slowly adding solid NaOH while monitoring with a pH meter accurate to 0.01 unit. Bring the volume to 1000 ml with Milli‐Q H_2_O and transfer the solution to a labeled storage bottle.

### Lysis working solution

**Table:** 

Reagent	Final concentration	Quantity or volume
Lysis stock solution (stored at 4°C).	–	98 ml
Triton X‐100 (ThermoScientific, cat. no. A16046.AE)	0.5%	1 ml
Dimethyl sulfoxide (DMSO)	0.5%	0.5 ml
Total		100 ml

Prepare at least 30 min before use. Remove the lysis stock solution (see recipe) from the 4°C refrigerator and place it on a magnetic stirrer. Add Triton X‐100 and DMSO. Stir gently until fully dissolved. Cool the solution to 4°C for 30 min before use. Use immediately after cooling. Do not store leftovers.

### Neutralization buffer, pH 7.5

**Table:** 

Reagent	Final concentration	Quantity or volume
Tris base	0.4 M	48.5 g
Milli‐Q‐purified (ultrapure) water	–	∼850 ml (adjust to 1 liter)
Total		1000 ml

Prepare at least 1 day before use. In ∼850 ml of Milli‐Q H_2_O on a magnetic stirrer, completely dissolve Tris base. Adjust the pH to 7.5 by adding HCl dropwise, monitoring the pH with a calibrated pH meter accurate to 0.01 unit. Once adjusted, bring the final volume to 1 L with Milli‐Q H_2_O and transfer to a clean bottle. Store up to 15 days at room temperature.

*Alternative method (preparation from commercial Tris*·*Cl solution)*: Add 400 ml Tris·Cl (1 M, pH 8.0) to a 1‐L container. Add 600 ml of Milli‐Q H_2_O and mix thoroughly. Adjust the pH to 7.5 by adding HCl dropwise while stirring. Monitor the pH with a calibrated pH meter accurate to 0.01 unit. Ensure the total volume is 1 L.

### PBS‐T, 0.1%

**Table:** 

Reagent	Final concentration	Quantity or volume
Tween 20 (Sigma‐Aldrich, cat. no. P9416; CAS no. 9005‐64‐5)	1×	500 µl
1× PBS, pH 7.4	–	500 ml
Total		500 ml

To prepare, add Tween‐20 to 1× PBS and mix thoroughly. Store at room temperature for up to 1‐2 weeks or prepare fresh as needed. Ensure the solution is well mixed before each use.

### Precoated microscope slides (1% NMP agarose)

**Table:** 

Reagent	Final concentration	Quantity or volume
UltraPure agarose (NMP)	1% (w/v)	0.2 g
Milli‐Q‐purified (ultrapure) water	–	20 ml
Total	–	20 ml

Prepare at least 1 day before. Weigh NMP agarose and dissolve it in Milli‐Q H_2_O by heating in the microwave, swirling every 30 s until the solution is clear. Keep the molten agarose at 60°C. Dip the frosted end of Superfrost Plus slides into the hot 1% NMP agarose and wipe the back to remove excess. Let the slides air‐dry horizontally for 1 hr or overnight in a dust‐free environment. Store up to 1 month in a dry place at room temperature.

### Primary antibody solution (anti‐BrdU)

**Table:** 

Reagent	Final concentration	Quantity or volume
Mouse monoclonal anti‐BrdU (clone B44; BD Bioscience, cat. no. 347580)	2.5 µg/ml	5 µl
1% BSA/PBS	–	495 µl
Total		500 µl

Prepare fresh on the day of use. Allow the mouse monoclonal anti‐BrdU antibody (clone B44) to reach room temperature if stored at 4°C. In a sterile 1.5‐ml microcentrifuge tube, combine 495 µl blocking buffer (1% PBS/BSA) with 5 µl of the antibody stock (1:100 dilution). Mix gently by pipetting up and down—do not vortex. Use immediately after preparation. For increased signal intensity, the antibody concentration can be raised to a 1:50 dilution (e.g., 10 µl antibody + 490 µl blocking buffer).

### Secondary antibody, Alexa Fluor 594 dye conjugated

**Table:** 

Reagent	Final concentration	Quantity or volume
Goat anti‐mouse IgG (H+L) secondary antibody conjugated to Alexa Fluor 594 (Invitrogen, cat. no. A‐11005)	50 µg/ml	1 µl
1% BSA/PBS	–	499 µl
Total		500 µl

Prepare fresh. Remove the Alexa Fluor 594‐conjugated secondary antibody from storage (typically at –20°C or 4°C) and allow it to equilibrate to room temperature if stored at 4°C. To a sterile 1.5‐ml microcentrifuge tube, add 499 µl blocking buffer (see recipe) followed by 1 µl of the secondary antibody stock (1:500 dilution). Mix gently by pipetting up and down—avoid vortexing to preserve antibody integrity. For enhanced signal detection, the antibody can be used at a 1:250 dilution (e.g., 2.5 µl antibody + 497.5 µl blocking buffer).

### SYBR Green staining solution

**Table:** 

Reagent	Final concentration	Quantity or volume
SYBR Green I nucleic acid gel stain (10,000× concentrate in DMSO; Life Technologies, cat. no. S11494)	1×	1 µl
Tris‐EDTA buffer	–	9999 µl
Total		10,000 µl

Prepare fresh on the day of use. In a sterile amber microcentrifuge tube (or a clear tube wrapped in foil), add 9999 µl of freshly prepared or prechilled 1× PBS. Carefully add 1 µl of SYBR Green I (10,000× stock) to obtain a 1:10,000 dilution. Mix gently by pipetting up and down—do not vortex. Always protect the solution from light. If not used immediately, store up to 6 months in a light‐protected container at room temperature.

## COMMENTARY

### Critical Parameters

#### Comet analysis

**Figure 3:**
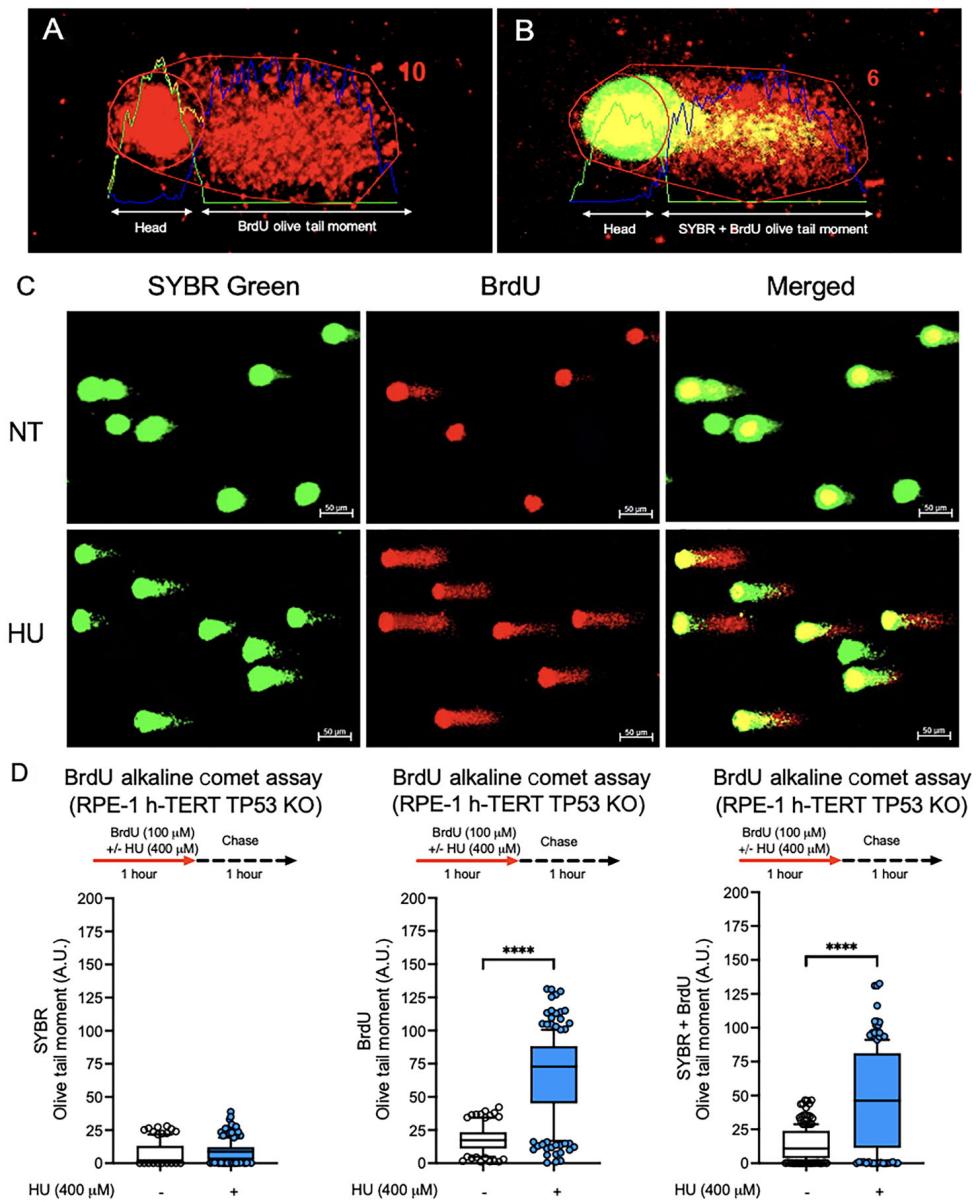
Detection, quantification, and validation of replication‐associated DNA damage using the alkaline BrdU comet assay in RPE‐1 h‐TERT TP53 KO cells. (**A and B**) Representative comet images demonstrating quantification methods: BrdU channel alone, used to measure replication‐associated Olive tail moment (OTM; **A**), and combined BrdU + SYBR Green channels, used to measure total DNA OTM (**B**). (**C**) Typical images of comets from RPE‐1 h‐TERT TP53 KO cells, either untreated (NT) or treated with 400 µM hydroxyurea (HU) for 1 hr, followed by a 1‐hr chase. SYBR Green (green) stains non‐replicated DNA, while BrdU immunofluorescence (red) marks newly replicated DNA. Merged images highlight co‐localization of BrdU and total DNA within comet tails. Scale bars, 50 µm. (**D**) Quantification of OTM in the alkaline comet assay for RPE‐1 h‐TERT TP53 KO cells. Left, SYBR‐only OTM; middle, BrdU‐only OTM; right, combined BrdU + SYBR OTM. HU treatment significantly increased DNA damage in newly replicated DNA, as indicated by BrdU‐only OTM. Statistical analysis was performed using Student's *t*‐test; ****p < 0.0001. Data include 200 comets (100 per condition) from two independent experiments (*n* = 2). Bars display the 10th to 90th percentile range, with the internal line indicating the mean; whiskers show minimum/maximum non‐outlier values; dots represent outliers. A.U., arbitrary units.

The alkaline BrdU comet assay allows accurate measurement of replication‐related DNA damage by distinguishing BrdU‐labeled DNA from the total genomic content. Selecting the appropriate comet type and fluorescence channel for analysis is crucial for precise interpretation (see Fig. 3).

#### Comet selection criteria

For quantification, include comets with visible and distinguishable BrdU and SYBR Green signals. As illustrated in Figure 3C, comets can vary in BrdU distribution:

● Type I: Strong BrdU staining in comet tail, ideal for replication‐associated DNA damage quantification.
● Type II: Overlapping BrdU and SYBR Green signals, reflecting total DNA damage.
● Type III: Predominantly head‐localized signal with little or no tail, often excluded from BrdU‐specific analysis.

#### BrdU‐specific Olive tail moment—replication‐associated DNA damage

Replication‐associated DNA damage is quantified by analyzing comets in the BrdU fluorescence channel only. This measurement (Fig. 3A and Fig. 3C, middle panel) reflects the migration of BrdU‐incorporated DNA under alkaline conditions, corresponding to replication‐associated DNA breaks. This parameter is particularly sensitive to replication stress induced by agents such as hydroxyurea (HU).

##### BrdU + SYBR Green Olive tail moment—total DNA damage

The total DNA damage is assessed by analyzing the combined BrdU and SYBR Green signals (Fig. 3B and Fig. 3C, right panel). This readout represents the global level of strand breaks across both replicated and non‐replicated DNA. However, replication‐specific lesions may be underestimated if BrdU incorporation is limited or uneven.

##### Comparative analysis/representations

Compile the quantified data and calculate the mean ± standard deviation (SD) for both technical and biological replicates. Use GraphPad Prism (or equivalent software) to create either bar graphs (showing mean ± SD) or scatter plots (displaying individual data points). Predefine the threshold for statistical significance (e.g., *p* < 0.05). Figure 3D demonstrates the quantitative difference between these two approaches in a pilot experiment with RPE‐1 h‐TERT TP53 KO cells. HU treatment significantly increased Olive Tail Moment when quantified in the BrdU channel alone, highlighting replication‐associated DNA damage. In contrast, the SYBR channel alone or in combination with BrdU and SYBR Green measurement captured total DNA damage, but diluted replication‐specific effects. Together, these complementary readouts provide both a targeted and comprehensive perspective of DNA damage.

##### Variations

Any of the following may be adjusted based on the experimental hypothesis.

1. Longer treatment and/or chase period:
• Decrease BrdU concentration (e.g., 10‐20 µM) and adjust the treatment compound accordingly.
2. Chase period:
• Adjust based on the compound's mechanism of action to minimize damage over time.
3. Cell viability (optional):
• Assess using 0.4% trypan blue with a Countess Cell Counter or Neubauer chamber.
• Proceed only if viability is ≥70% to avoid false positives caused by excessive cell death.
4. Agarose and environmental conditions:
• The thickness of the agarose layer may alter the length of subsequent steps.
• Control environmental factors such as humidity and temperature.
• Protect samples from light whenever required.
5. Lysis step:
• Pre‐label slides before placing them in the Coplin jar.
• Optimize lysis times (1‐24 hr). If “halos” appear or comets are not observed in positive controls, consider extending the lysis to overnight (12‐24 hr).
• Before alkaline unwinding, briefly rinse slides with cold (4°C) 1× PBS to remove residual lysis buffer.
6. Electrophoresis and unwinding:
• Maintain the buffer temperature between 4°C and 10°C using an ice bath, cooling system, or cold room.
• Run electrophoresis for 20‐30 min.
• Ensure the buffer fully covers the slides and avoid any movement of the tank during electrophoresis.
• Minimize light exposure.
• For alkaline unwinding, limit the step to 20‐40 min to prevent excessive DNA degradation.
7. Neutralization:
• If a neutralization buffer is unavailable, use 1× PBS (pH 7.4) and cover with aluminum foil to shield from light.
• After neutralization, rinse with distilled water to remove any residual salts.
8. Fixation:
• Dehydrating slides with 99% ethanol before air drying helps keep comets in the same focal plane.
• Ethanol can be reused, but it must be stored appropriately to prevent evaporation.
• Once fixed, slides do not need to be kept in the dark.
• Ensure slides are fully dehydrated and free of dust‐free before storage.
9. Mini‐gels and coverslips:
• When using mini‐gels, avoid bubbles and ensure proper solidification.
• Do not add an agarose layer before enzyme incubation, as it can interfere with diffusion.
10. Trypsinization:
• Avoid overexposure to trypsin to prevent additional DNA damage and background signal.
• Consider using TrypLE, Accutase, or gentle scraping as alternatives means of cell detachment.
11. Immunostaining:
• If background staining is high, increase blocking time or dilute the secondary antibody to reduce non‐specific binding.
12. Weak BrdU signal:
• Confirm that DNA denaturation was effective.
• Maintain high slide humidity to prevent agarose gel drying and loss of antibody activity.
13. Staining and imaging:
• Perform all steps in a location away from direct light to prevent photobleaching.
• Avoid analyzing overlapping nuclei or comets located near the gel edges.
• Rinse slides thoroughly with Milli‐Q‐purified water to remove excess dye.
• Adjust imaging settings to avoid signal loss or overexposure.
14. Data consistency and analysis:
• Ensure that scoring remains consistent across all experimental groups to minimize operator bias.
• Verify automated image analysis tools before using them regularly.
15. Limitations:
• This protocol is optimized for mammalian adherent and suspension cells. It primarily detects replication‐associated DNA damage and may require further adjustments for other cell types or experimental systems.

### Troubleshooting

Table 1 discusses common problems with the protocol, their causes, and potential solutions.

**Table 1:** Troubleshooting for the Alkaline BrdU Comet Assay

Problem	Cause	Solution
Mini‐gel instability or bubbles	Incomplete solidification or an additional agarose layer interfering with enzyme diffusion	Ensure proper solidification and avoid bubbles. Do not apply additional agarose layers if enzyme incubation is required.
Halos in nucleoids or absent comets in positive controls	Incomplete lysis	Perform overnight lysis (12‐24 hr) for complete removal of cellular components. Rinse slides briefly with Milli‐Q‐purified water before alkaline unwinding.
Increased DNA damage in all samples (including NT controls)	Possible overexposure to trypsin	Use TrypLE, Accutase, or gentle scraping as alternatives to minimize background signal.
Uneven electrophoresis migration (distorted comets)	Inconsistent electrophoresis conditions or buffer evaporation.	Maintain a stable temperature (4°C) using a cooling system or cold room. Ensure that the buffer level fully submerges slides, and standardize voltage and duration across experiments.
High background signal in immunostaining	Nonspecific secondary antibody binding or inadequate blocking	Extend blocking time with 1% BSA/PBS before primary (anti‐BrdU) antibody incubation. Use a higher dilution of the secondary antibody and ensure thorough washing between steps.
Weak or absent BrdU staining signal in treated samples	Inefficient DNA denaturation	Optimize the alkaline step by ensuring sufficient incubation time. Confirm antibody specificity. Maintain slide humidity to prevent gel drying.
Comets with excessive DNA fragmentation	Overly harsh lysis or excessive alkaline unwinding	Adjust lysis duration or detergent concentration in lysis buffer. Decrease alkaline unwinding time to 20‐30 min at 4°C.
Low percentage of cells forming comets	Improper embedding of cells in agarose or poor cell suspension preparation	Maintain LMP agarose suspension at 37°C before applying cells to avoid air bubbles and overheating.
Poor comet visualization under fluorescence microscopy	Inadequate immunostaining or excessive background fluorescence	Use freshly staining solutions. Rinse and mount slides before imaging. Optimize exposure time in fluorescence microscopy.
Staining degradation affecting comet visualization	Exposure to direct light	Perform staining steps away from direct light to prevent fluorescence degradation.
Artifacts in image analysis (distorted comet morphology)	Edge effects from gel preparation	Avoid analyzing comets near the edges of gels, as their morphology may be distorted.
Data inconsistency between replicates	Variability in sample handling or cell damage	Standardize cell collection, lysis, and electrophoresis procedures. Use duplicate gels/slides per condition and independent experiments for reproducibility.
Bias in comet scoring and analysis	Operator variability or software limitations	Score comets consistently across experimental groups. Validate automated image analysis tools to ensure accurate detection of damaged cells.
Protocol limitations for specific cell types	Optimization required for non‐mammalian cells	Adjust lysis and electrophoresis conditions when working with non‐mammalian cells.

### Statistical Analysis

1. An Excel template can be created to calculates descriptive statistics for each sample, including mean, median, and standard deviation (SD). Data can be shown separately for standard comets and all comets, including outliers, depending on the experimental criteria.
2. For data visualization, use either bar graphs or scatter plots. (a) Bar graphs: Plot the mean Olive tail moment (± SD) for each biological replicate. Show BrdU‐only and BrdU + SYBR Green data in separate graphs. (b) Scatter plots: Display individual BrdU Olive tail moment values for at least 200 nucleoids per treatment, ensuring data from at least two independent experiments for each condition.
3. For statistical analysis, use an unpaired Student's *t‐*test for comparisons between two groups (e.g., NT vs. HU‐treated). For three or more groups, use one‐way ANOVA followed by Bonferroni's post hoc test (or a suitable alternative such as Tukey's post hoc HSD test). Verify assumptions of normality, or apply non‐parametric tests (e.g., the Mann‐Whitney *U* test or the Kruskal‐Wallis test) if needed.
4. When publishing comet assay data, it is advisable to follow the Minimum Information for Reporting Comet Assay (MIRCA) guidelines (Mooler et al., 2020), which promote transparency, reproducibility, and consistency across studies.
5. When applicable, briefly explain the rationale behind the chosen statistical approach and discuss alternative methods if they are more appropriate for the data distribution or experimental design.

### Understanding Results

The alkaline BrdU comet assay was established as a sensitive and specific method for quantifying replication‐associated DNA damage at the single‐cell level. This assay combines BrdU pulse labeling with the alkaline comet assay protocol and immunodetection to selectively measure DNA breaks in nascent DNA. Figures 1 and 2 provide a step‐by‐step overview of the protocol, from cell treatment and BrdU incorporation to image acquisition and quantification, serving as a visual reference for users to benchmark their implementation of the method. To illustrate how replication‐associated DNA damage is identified, representative images are shown in Figure 3. SYBR Green (green) stains non‐replicated DNA, while incorporated BrdU in nascent DNA is detected in red, making it possible to distinguish between replicating and non‐replicating DNA. Co‐localization of both signals in the comet tail (yellow in merged images) indicates replication‐dependent DNA damage. This classification enables a clear distinction between general DNA breaks and those specifically occurring during DNA replication, thus improving the accuracy of assessing genotoxic stress in S‐phase cells. Validation experiments using RPE‐1 h‐TERT TP53 KO cells treated with hydroxyurea (HU), a ribonucleotide reductase inhibitor that causes replication stress by depleting dNTPs and stalling replication forks, demonstrated the assay's ability to distinguish replication‐associated DNA damage from total genomic breaks (Fig. 3D). HU treatment resulted in a significant increase in Olive tail moment (OTM) when specifically quantified in the BrdU channel, highlighting replication‐stress‐induced breaks. In contrast, combined BrdU + SYBR analysis reflected overall DNA damage but partially masked replication‐specific effects. For completeness, no higher tails were observed when OTM was evaluated only in the SYBR channel, demonstrating that the breaks in red comets are specific to replicating DNA quantification. This result suggests that BrdU pulse‐labeling detection is highly sensitive and can detect very small fragments that SYBR Green cannot identify, at least under the conditions used in this study.

**Figure 4:**
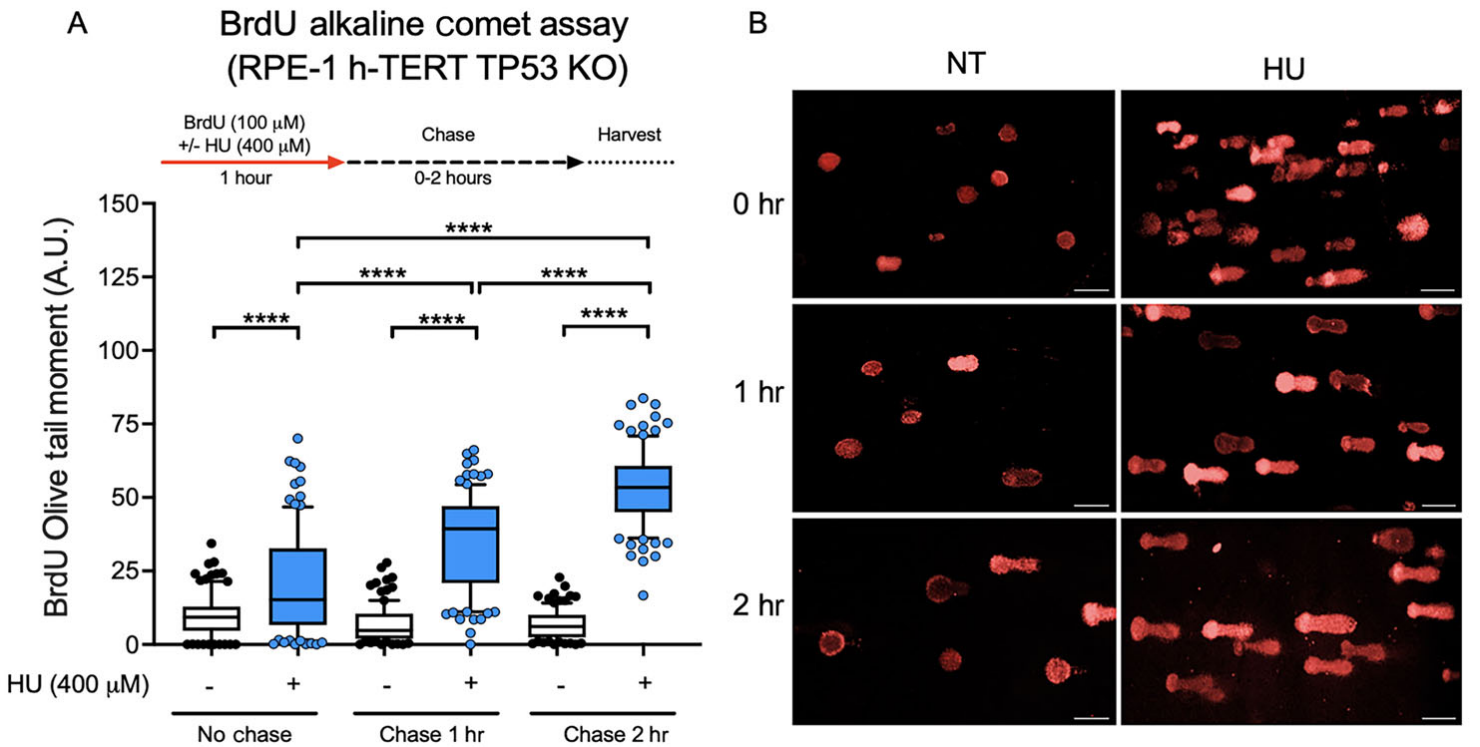
Replication‐associated DNA damage in RPE‐1 h‐TERT TP53 KO cells following HU treatment. Cells were labeled with BrdU (100 µM) and either co‐treated with HU (400 µM) or not for 1 hr, and then harvested immediately (no chase) or after 1 or 2 hr of chase in BrdU/HU‐free medium. (**A**) Quantification of BrdU Olive tail moment (in arbitrary units, A.U.) in untreated (NT) and HU‐treated cells. HU significantly increased replication‐associated DNA damage at all time points. Statistical analysis was performed using one‐way ANOVA followed by Bonferroni's multiple comparison test; *p < 0.05; **p < 0.01; ***p < 0.001; ****p < 0.0001. (**B**) Representative BrdU‐stained comets (red) from NT and HU‐treated cells at each chase period. Each condition includes 200 comets analyzed from two independent experiments (*n* = 2). Bars show the 10th to 90th percentile range, with the internal line representing the mean; whiskers display minimum and maximum non‐outlier values, and dots represent outliers. Scale bars, 50 µm.

**Figure 5:**
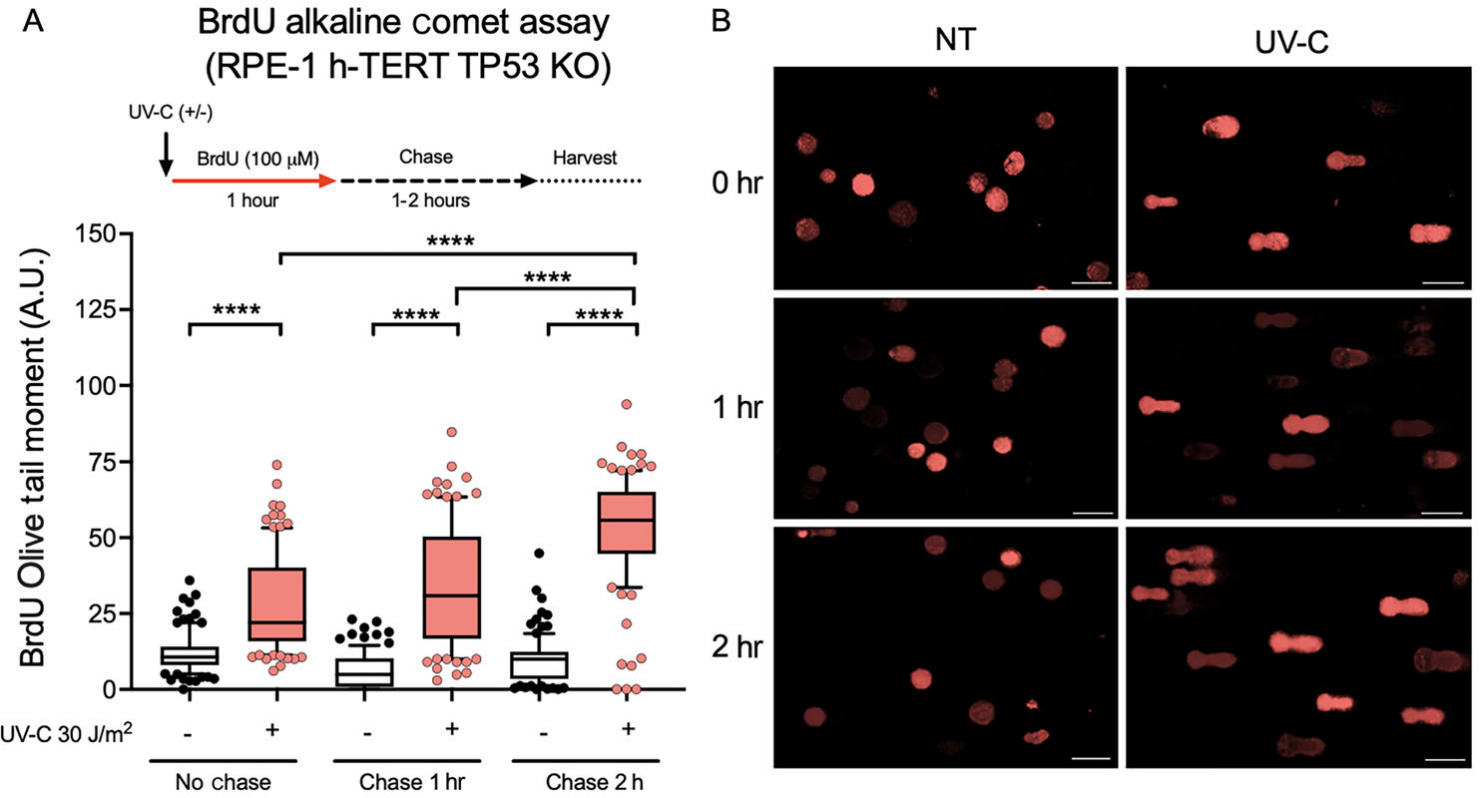
Replication‐related DNA damage in RPE‐1 h‐TERT TP53 KO cells after UV‐C exposure. Cells were either exposed or not exposed to UV‐C (30 J/m^2^), pre‐labeled with BrdU (100 µM) for 1 hr, and then harvested immediately (no chase) or after 1 or 2 hr of chase in BrdU‐free medium. (**A**) Bars showing BrdU Olive tail moment (in arbitrary units, A.U.oli) in untreated (NT) and UV‐C‐treated cells. UV‐C significantly increased replication‐related DNA damage at all time points. Statistical analysis was conducted using one‐way ANOVA followed by Bonferroni's multiple comparison test; *p < 0.05; **p < 0.01; ***p < 0.001; ****p < 0.0001. (**B**) Representative BrdU‐stained comets (red) from NT and UV‐C‐treated cells at each chase period. Each condition includes 200 comets (100 nucleoids per condition) analyzed from two independent experiments (*n* = 2). Bars show the 10th to 90th percentile range, with the internal line representing the mean; whiskers display minimum and maximum non‐outlier values, and dots represent outliers. Scale bars, 50 µm.

In Figure 4, BrdU‐labeled RPE‐1 h‐TERT TP53 KO cells were exposed to 400 µM HU for 1 hr, followed by no chase (0‐hr chase) and chase periods of 1 and 2 hr. As expected, there was a significant increase in BrdU OTM in HU‐treated cells compared to untreated controls (Fig. 4A), indicating that replication forks collapsed under HU‐induced stress. Representative comet images (Fig. 4B) visually support the quantification, showing that HU‐treated cells have longer comet tails and more intense BrdU signals. These results confirm that the assay reliably detects replication‐associated DNA damage and can serve as a benchmark for evaluating genotoxic agents and replication stressors. Interestingly, there appears to be an increase in BrdU‐labeled DNA breaks after 1‐ and 2‐hr chase periods, suggesting that replication stress persists even after treatment, possibly because of discontinuities in replicating DNA. We further extended the assay to compare the kinetics of replication stress in RPE‐1 TP53 KO cells exposed to UV‐C (30 J/m^2^) radiation or no treatment. Cells were incubated with BrdU for 1 hr after irradiation, and, as shown in Figure 5, DNA damage was measured at 0 (after irradiation/no chase), 1, and 2 hr of chase. The BrdU OTM increased significantly following UV‐C irradiation at all time points, with the highest damage observed at 2‐hr chase (Fig. 5A), also indicating that replicating DNA is accumulating discontinuities. Representative images (Fig. 5B) show the gradual buildup of comet tails, characterized by a strong BrdU signal, especially in 2‐hr chase‐treated samples. These results demonstrate that the BrdU comet assay can detect dynamic changes in replicated DNA damage over multiple time points.

**Figure 6:**
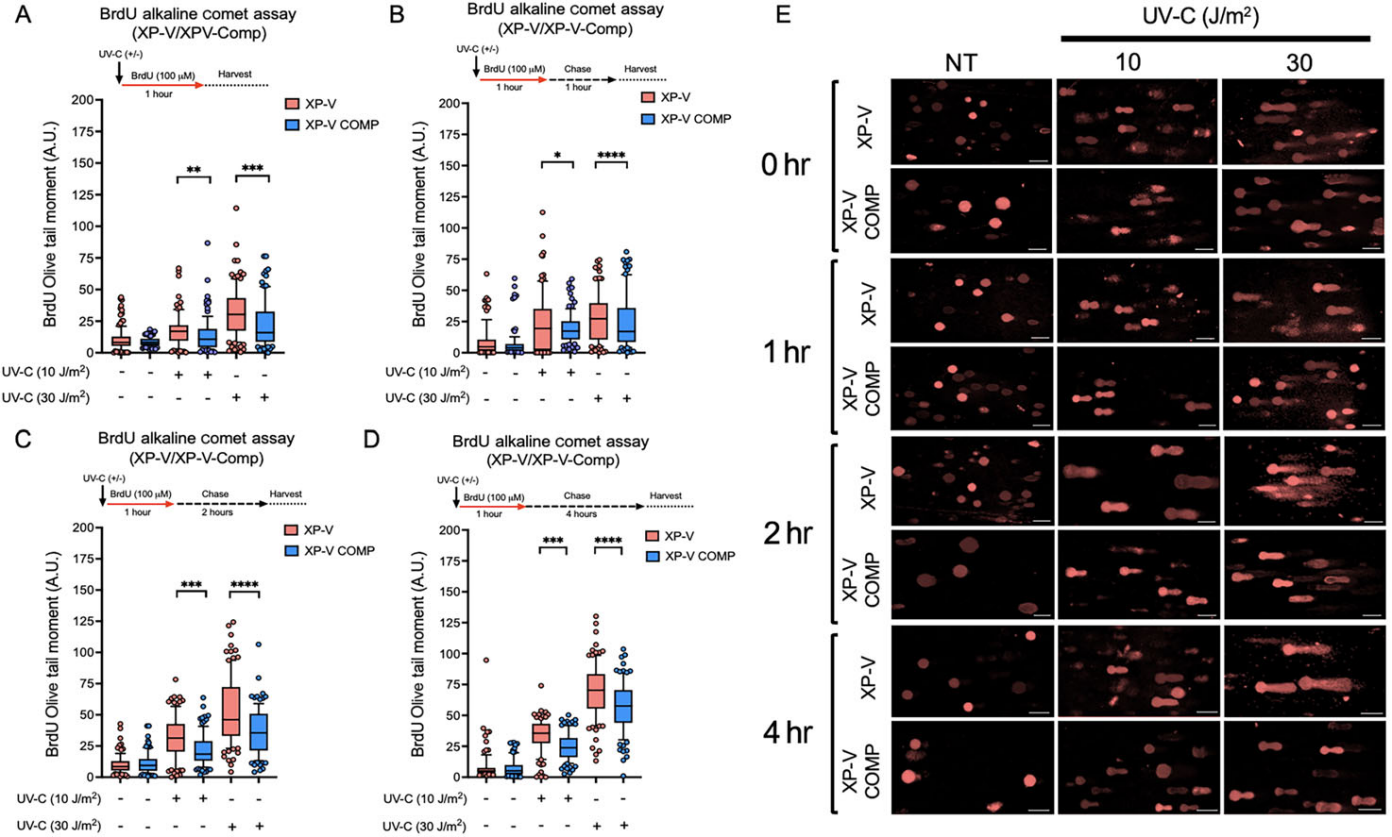
XP‐V cells exhibit increased replication‐related DNA damage compared to complemented cells after UV‐C irradiation. Alkaline BrdU comet assay was performed on XP‐V and XP‐V complemented (XP‐V Comp) cells after UV‐C irradiation (10 or 30 J/m^2^) and BrdU pulse (100 µM), followed by chase periods or immediate harvest as indicated. BrdU Olive tail moment was measured (**A**) immediately after BrdU pulse (no chase period), (**B**) 1 hr after BrdU pulse (1‐hr chase), (**C**) 2 hr after BrdU pulse (2‐hr chase), and (**D**) 4 hr after BrdU pulse (4‐hr chase). In all conditions, XP‐V cells exhibited significantly higher BrdU tail moments than XP‐V Comp cells, especially after higher UV‐C doses and longer chase times, indicating impaired repair of replication‐associated damage. A.U., arbitrary units. (**E**) Representative comet images showing BrdU staining (red) in XP‐V and XP‐V Comp cells under various conditions and time points. UV‐C‐induced comet tails are longer in XP‐V cells, consistent with increased DNA damage and replication stress. Scale bars, 50 µm. Each condition includes 200 comets (100 nucleoids per condition) analyzed from two independent experiments (*n* = 2). Bars show the 10th to 90th percentile range, with the internal line representing the mean; whiskers display minimum and maximum non‐outlier values, and dots represent outliers. Statistical analysis was performed using a two‐way ANOVA with Bonferroni's multiple comparison test; *p < 0.05; **p < 0.01; ***p < 0.001; ****p < 0.0001.

To demonstrate the assay's usefulness in measuring PRR, we examined the effects of different doses of UV‐C irradiation on human xeroderma pigmentosum variant (XP‐V) fibroblast cells and their complemented counterparts (XP‐V Comp). As shown in Figure 6, XP‐V cells, which lack the TLS Polη, accumulated significantly higher BrdU OTMs compared to XP‐V Comp cells, especially at 30 J/m^2^. This difference was evident immediately after the BrdU pulse (Fig. 6A) and persisted during 1‐, 2‐, and 4‐hr chase periods (Fig. 6B‐D), indicative of delayed or impaired processing of replication‐blocking lesions in the absence of functional TLS, a DDT mechanism. Again, the increase in DNA damage in both cell lines suggests the increase in discontinuities in replicating DNA. The quantification is supported by representative comet images (Fig. 6E), in which XP‐V cells display prominent BrdU‐positive comet tails, particularly at higher UV‐C doses and longer chase times. These findings demonstrate the assay's effectiveness in detecting timing deficiencies in replication‐associated DNA damage. The higher BrdU OTM observed in TLS‐deficient XP‐V cells after UV‐C exposure, compared to complemented cells, indicates increased replication‐associated DNA damage, such as ssDNA gaps and strand breaks in newly synthesized DNA. Although XP‐V cells are proficient in nucleotide excision repair (NER) and can eventually remove UV‐induced cyclobutane pyrimidine (CPD) dimers and 6‐4 photoproducts, the lack of TLS Polη prevents efficient lesion bypass during the S phase. This results in a temporary vulnerability period in which replication forks stall or form gaps until NER repairs the damage. By analyzing BrdU comet parameters at various chase times after UV‐C treatment, researchers can track the PPR and/or DDT, revealing the dynamic interaction between the lesion removal and TLS‐dependent replication fork progression.

Overall, the alkaline BrdU comet assay provides sensitive detection and quantification of replication‐associated DNA damage, serving as a powerful tool for studying replication stress, fork collapse, DDT, and PRR capacity. In our experience, the assay not only successfully distinguished between different replication‐associated DNA damage and repair phenotypes but also uncovered additional details in DNA damage tolerance kinetics, emphasizing its usefulness for analyzing the interaction between lesion bypass and DNA repair pathways. Its application to both pharmacological and genetic models demonstrates its robustness and reproducibility, offering a clear standard for users following the protocol. Adherence to MIRCA recommendations enhances assay reliability and supports the integration of results into the broader International Comet Assay Working Group (ICAW) initiatives.

### Time Considerations

Basic Protocol can be finished within 6‐8 hr on day 1, including treatments, BrdU pulse, and electrophoresis, plus ∼4 hr on day 2 for immunostaining and imaging. With slide preparation on day 0, the overall duration is 3 days. Alternate Protocol 1 requires a similar schedule, but chase periods (0‐2 hr) extend the handling time on day 1. The total duration is also 3 days. Alternate Protocol 2 includes more extended chase periods (0‐4 hr) and therefore extends day 1 to ∼8‐9 hr. Day 2 requires ∼4 hr for staining and imaging, resulting in an overall duration of 3 days.

### Author Contributions

**Diego Luis Ribeiro**: Conceptualization; methodology; investigation; data curation; formal analysis; visualization; writing—original draft; writing—review and editing. **James Eduardo Lago Londero**: Methodology; investigation; writing—original draft; writing—review and editing. **Davi Jardim Martins**: Methodology; investigation; data curation; formal analysis; visualization; writing—original draft; writing—review and editing. **Carlos Frederico Martins Menck**: Conceptualization; methodology; writing—review and editing; supervision; funding acquisition. All authors approved the final manuscript.

### Conflict of Interest

The authors declare no conflict of interest.

## Data Availability

The data utilized in this study can be found in the article.
